# Penile cancer in Maranhão, Northeast Brazil: the highest incidence globally?

**DOI:** 10.1186/s12894-018-0365-0

**Published:** 2018-05-29

**Authors:** Ronald Wagner Pereira Coelho, Jaqueline Diniz Pinho, Janise Silva Moreno, Dimitrius Vidal e Oliveira Garbis, Athiene Maniva Teixeira do Nascimento, Joyce Santos Larges, José Ribamar Rodrigues Calixto, Leandra Naira Zambelli Ramalho, Antônio Augusto Moura da Silva, Leudivan Ribeiro Nogueira, Laisson de Moura Feitoza, Gyl Eanes Barros Silva

**Affiliations:** 1 Aldenora Bello Cancer Hospital, Seroa da Mota Street, Apeadouro, São Luís, 65031-630 Brazil; 2 Federal University of Pará, Brazil, Gov. José Malcher Avenue, Belém, 66055-260 Brazil; 3 Federal University of Maranhão, São Luís, Brazil, dos Portugueses Avenue, Bacanga, São Luís, 65080-805 Brazil; 4 University Hospital of Federal University of Maranhão, Barão de Itapari Street, Centro, São Luís, Brazil; 5 Department of Radiology and Pathology, Ribeirão Preto Medical School of University of São Paulo, Bandeirantes Avenue, Monte Alegre, Ribeirão Preto, 14049-900 Brazil; 6 Public Heath Departament, Federal University of Maranhão, São Luís, Brazil, dos Portugueses Avenue, Bacanga, São Luís, 65080-805 Brazil; 7 Ribeirão Preto Medical School - USP, Av. Bandeirantes, 3900, Ribeirão Preto, SP 14048-900 Brazil

**Keywords:** Carcinoma, Penis cancer, Age-standardized incidence, Penectomy, Squamous cell carcinoma

## Abstract

**Background:**

The objectives of this study were to determine the minimum incidence of penile cancer in the poorest Brazilian state, and to describe the epidemiologic and clinical characteristics of patients diagnosed with the disease.

**Methods:**

A retrospective study of 392 patients diagnosed with penile cancer in the three most important referral center in the state was conducted during 2004–2014.

**Results:**

The age-standardized incidence was 6.15 per 100,000 and the crude annual incidence was 1.18 per 100,000. More than half (61.1%) of the tumors were histological grades 2 and 3, and 66.4% of tumors were classified as at least stage T2. The average age of patients was 58.6 ± 15.7 years (range, 18 to 103 years), with 20.8% of patients ≤40 years of age at diagnosis. The vast majority underwent penectomy (93%). Only 41.8% underwent lymphadenectomy, 58 patients (14.8%) received chemotherapy, and 54 patients (13.8%) received radiotherapy. Stage 3/4 and vascular invasion were statically significant at disease-free survival analysis.

**Conclusion:**

The state of Maranhão has the highest incidence of penile cancer in Brazil and globally. Tumors are locally advanced and at the time of diagnosis, and there is a high frequency among young individuals. Patients have a low socioeconomic status, making it difficult to complete treatment and receive appropriate follow-up.

## Background

Penile cancer is a rare neoplasm in developed countries. However, the incidence in developing countries in Asia, Africa, and Latin America is high, accounting for up to 10% of malignant neoplasms in men [1–3]. Brazil stands out among the countries with the highest incidences of penile cancer in the world, although no reliable data exist [4, 5]. In Brazil, the condition may account for 2.1% of all neoplasias in men, and affects mainly inhabitants of the North and Northeast regions [6]. Among the known risk factors for the development of penile cancer, poor hygiene, phimosis, human papillomavirus (HPV) infection, use of tobacco, and risky sexual behavior, are the most highlighted [7–10]. The disease mainly affects individuals with low socioeconomic levels and low levels of education [11–15].

Maranhão is the most rural state in Brazil, being historically marked by great social inequality and extreme poverty. Access to health care in the rural parts is poor, which leads the male population to seek medical attention in the capital city only when the disease is at an advanced stage. According to the Ministry of Health, during the period 1992–2007, 6716 penectomies were performed in Brazil, out of which 419 (6.2%) in the state of Maranhão. These data, albeit alarming, are underestimated and insufficient for understanding the reality of penile cancer in Maranhão. Hence, the objectives of this study were to estimate the minimum incidence of penile cancer in the state of Maranhão, and to describe the epidemiologic and clinical characteristics of patients diagnosed with the disease, in order to provide the basis for the development of measures to confront this disease and to act as an impetus for future studies.

## Methods

A retrospective cohort study was conducted. The studied sample was of composed of 286 patients newly diagnosed with penile cancer from January 2004 to December 2014 at the Cancer Hospital Aldenora Bello (HCAB). HCAB is a high complexity oncology center in Maranhão and the main referral center for the treatment of penile cancer in the state, which has an estimated population of 6,680,884 inhabitants, according to data from the Brazilian Institute of Geography and Statistics (IBGE).

Data were collected from physical and electronic records of all patients classified by the Hospital Cancer Record of HCAB as having penile cancer. The studied variables were age, marital status, education, occupation, home town, tumor location, histological type and grade, tumor size, type of surgery, lymphadenectomy, staging (TNM system, 2010), chemotherapy, and radiotherapy. Data were stored in Microsoft Excel spreadsheet editor and analyzed using Stata statistical software (Version 7.0, StataCorp, College Station, Texas).

Frequencies and percentages were used to express categorical variables, while numerical variables were presented as means and standard deviations. Age-standardized incidence rate (ASR) were calculated using the standard world population proposed by Segi and modified by Doll et al. [16] This method was also applied in the *Cancer Incidence in Five Continents* series of the International Agency for Research on Cancer (IARC), wherein the number of cases, in each 5-year age-stratum was divided by the population size in each age group [16]. In addition, due to the unavailability of ASR data in studies conducted in Brazil, we calculated an estimated annual crude incidence rate in our sample, in order to compare with data from previous Brazilian studies. Since there are others small centers that receive patients with penile cancer in Maranhão, the incidence estimated from the data herein therefore provides only a minimum estimate.

Survival analysis was performed using the Kaplan-Meier method to determine disease-free survival. The Log Rank test was used to compare survival curves. The significance threshold use was *p* ≤ 0.05. This work was in accordance with the principles of the Declaration of Helsinki and approved by the Research Ethics Committee of the Universidade Federal do Maranhão [process no. 43774215.7.0000.5086] and informed consent was waived.

## Results

From 2004 to 2014, 392 patients were diagnosed with penile cancer, resulting in an average of 36 cases per year. This translates to an ASR of 13.89 per 100,000 men over an 11-year period, corresponding to an average ASR of 6.15 per 100,000 men over a 5-year period. The crude incidence rate was 1.18 per 100,000 men per year. Patients from rural areas comprised 82.1% of the sample (322/392). Most were farmers (71%) and most (66,3%) reported being married. The average age of the studied sample was 58.6 ± 15.7 years (range 18 to 103 years), with 19.7% of patients aged ≤40 years at diagnosis. The majority of our patients 29% (72/248) were non-swokers.

All patients were diagnosed with squamous cell carcinoma. Among 136 the cases with reviewed histological subtype, according to most recent WHO classification (2016), 91 (66.9%) were of the subtypes of cancer that are associated with the presence of HPV, which include warty, basaloid, or mixed carcinomas with warty or basaloid components. More aggressive subtypes such as basaloid or sarcomatoid carcinoma was observed in 13 cases (9.6%) and 01 case (0.7%), respectively. The primary location of the tumor was the glans in 74.0% (158/212216/292) of cases. More than half (66.1%) of the tumors were histological grades 2 and 3. Using the TNM classification system, 66.4% (157/246) of tumors were classified as ≥T2. Lymph node involvement was present in 54% (93/175) of patients, and was distributed as follows: N1 in 8.6%, N2 in 28.7%, and N3 in 16.7%. Distant metastases were detected in 9 of the 138 patients for whom it was possible to search for metastatic disease (Table 1).

**Table 1:** Histologic features and staging of patients with penile cancer

	% (N)
Histological subtype	
Epidermoid carcinoma	100 (392/392)
Initial anatomic site	
Glans	74.0(216/392)
Undetermined^a^	15.0 (44/392)
Foreskin	11.0 (32/392)
Histologic grade	
Grade 1	38.9 (123/316)
Grade 2	54.1 (171/316)
Grade 3	7.0 (22/316)
Primary tumor (T stage)	
Tis	0.6 (2/335)
T1(a, b)	33.0 (107/335)
T2	44.6 (145/335)
T3	17.5 (57/335)
T4	4.3 (14/335)
Regional lymph nodes (N stage)	
N0	46.0 (107/233)
N1	8.6 (20/233)
N2	28.7 (67/233)
N3	16.7 (39/233)
Distant metástasis	
M0	45.9 (180/392)
M1	2.3 (9/392)
Mx	51.8 (203/392)
Staging	
Stage 0	26.0 (95/366)
Stage 1	30.0 (110/366)
Stage 2	26.5 (97/366)
Stage 3	15.3 (56/366)
Stage 4	2.2 (8/366)

^a^advanced destructive lesions

In terms of surgical procedures performed, the vast majority of patients (93%) underwent penectomy (210/279): 74.7% partial, 17.3% total, and 1% complete emasculation. Lymphadenectomy was performed in 41.8% (164/392) of patients overall; 64.2% underwent bilateral lymphadenectomy and 34.8% underwent unilateral lymphadenectomy. Chemotherapy was administered to 21.9% (54/246) of patients. Of these, 65% received chemotherapy as palliative therapy, and 3.6% as adjuvant therapy. Almost all (92.3%) patients who received chemotherapy had presented with advanced disease. Cisplatin and 5-fluorouracil were the most frequently used chemotherapeutic agents. Radiotherapy was used in the treatment of 27.1% (54/199) of patients, being adjuvant in 10 cases and palliative in the others. The radiation dose ranged from 20.2–60 Gy, with a dose of 50 Gy being most common (Table 2).

**Table 2:** Treatment modalities in patients with penile cancer

	% (N)
Type of surgery	
Partial penectomy	74.7 (286/383)
Total penectomy	17.3 (66/383)
Emasculation	1.0 (4/383)
Others (postectomy, glansectomy and excision)	7.0 (27/383)
Lymphadenectomy	
Yes	41.8 (164/392)
No	58.2 (228/392)
Chemotherapy	
Yes	23.6 (58/246)
No	76.4 (334/246)
Radiotherapy	
Yes	21.9 (54/246)
No	78.1 (145/246)

**Fig. 1:**
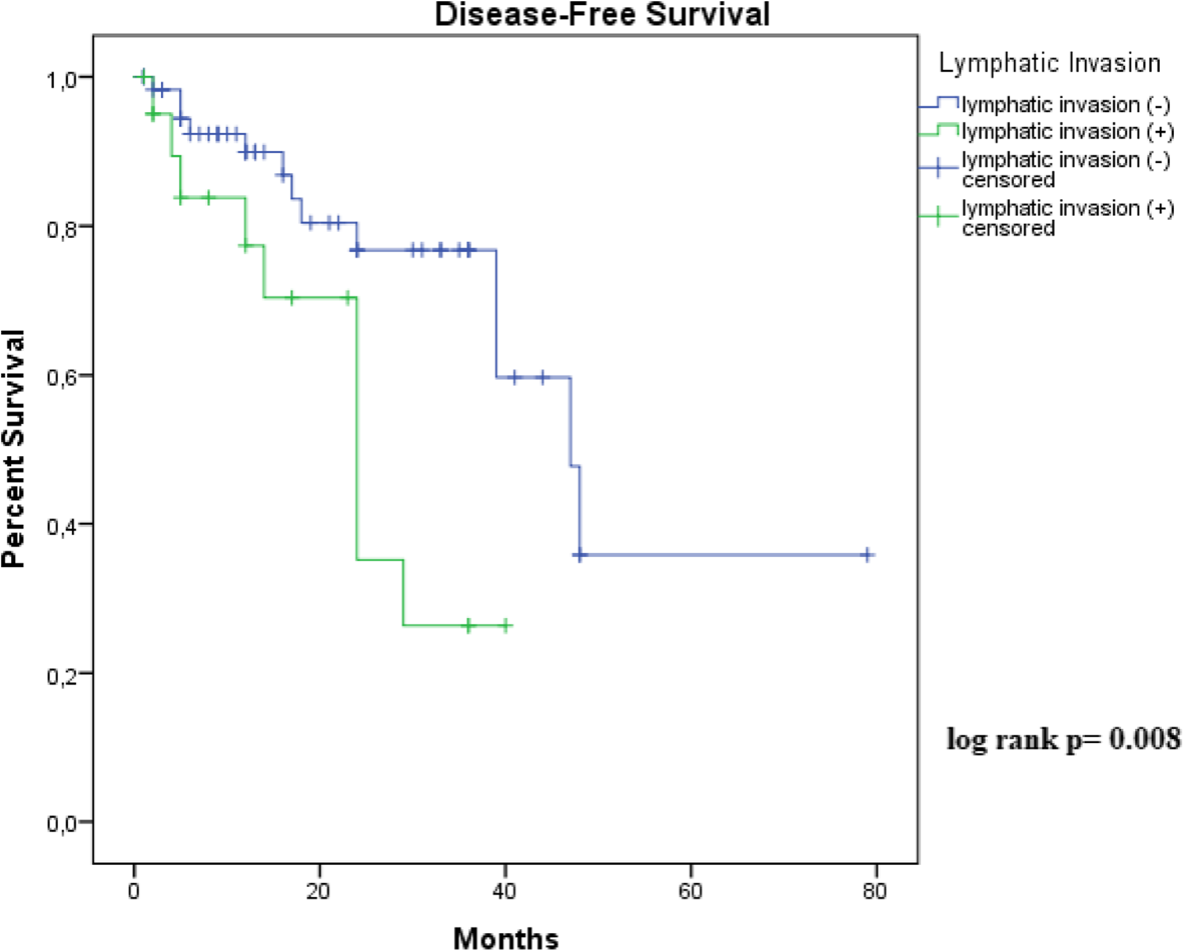
Kaplan-Meier estimated disease-free survival rate in available 111 patients according to American Joint Committee on Cancer (AJCC) stage (*p* ≤ 0.05)

**Fig. 2:**
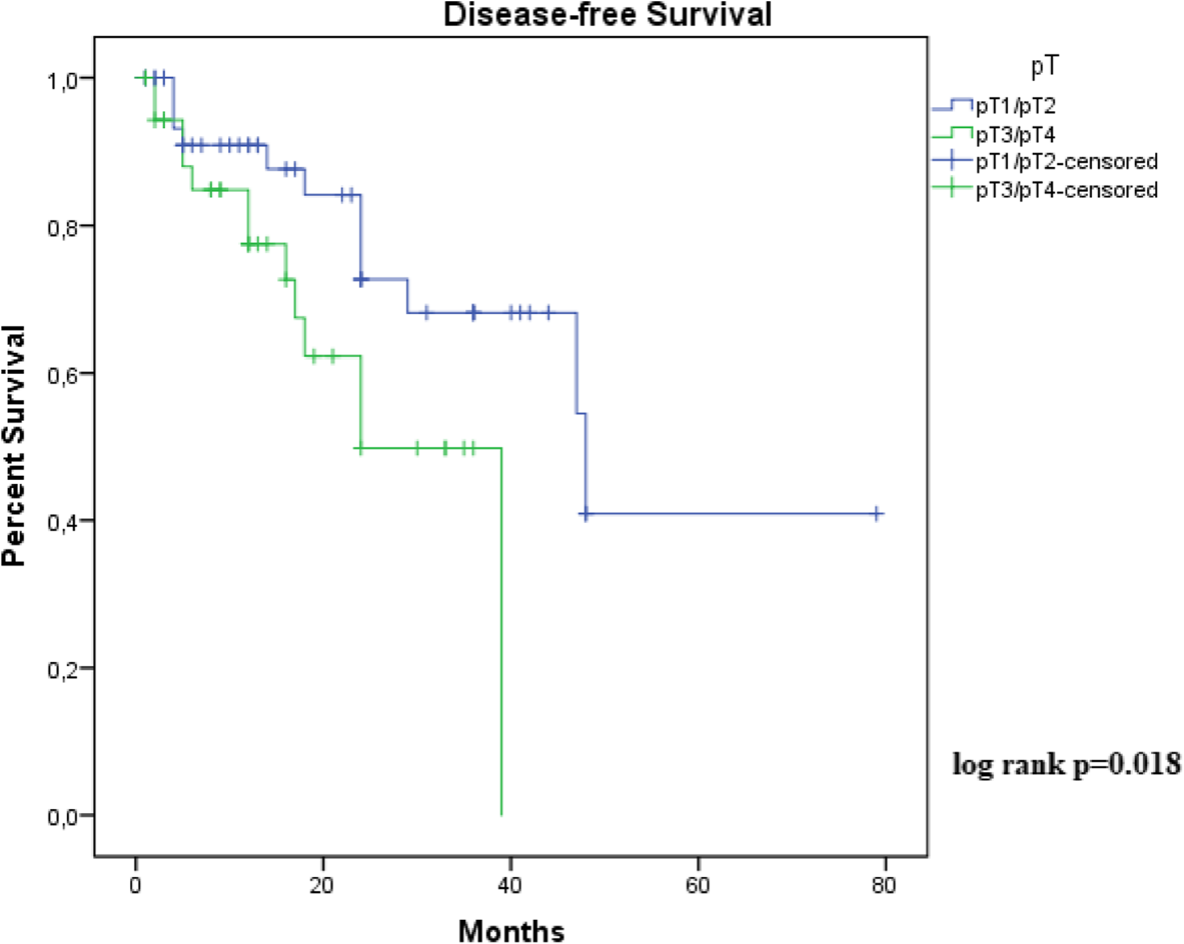
Kaplan-Meier estimated disease-free survival rate in available 111 patients according to presence of vascular invasion (*p* ≤ 0.05)

As shown in Figs. 1 and 2, Kaplan-Meier curves demonstrated that stage 3/4 and vascular invasion were associated with decreased disease-free survival (recurrence, Lymph node or systemic metastasis). Histological grade, tumor thickness, perineural invasion and tumor subtype were not significant predictors for disease-free disease. Due to great number of patients without follow-up, only 111 cases were included in this analysis. Follow-up ranged from 01 to 39 months (median 13,4 months). There was insufficient data on mortality for overall survival analysis.

## Discussion

The ASR of 6.1 per 100,000 men (5-year interval) recorded in this study exceeds the highest international rates reported by IARC (Table 3) [16]. No previous Brazilian study used age-adjusted or age-standardized incidence rates. In these prior studies, only the cumulative incidence rates were reported for different time intervals, making it necessary to calculate the annual average for comparison. The annual crude incidence rate in the present study was 1.18 per 100,000 men, far higher than any previously reported national annual crude incidence rate (Table 4) [6, 12, 17, 18].

**Table 3:** Highest age-standardized incidence rates of penile cancer in geographical areas of the world

Population	Age-standardized incidence (per 100,000)
Brazil, Maranhão	6.1
Brazil, Goiania	3.3
USA, Montana: American Indian	2.8
Brazil, Aracaju	2.7
Malawi, Blantyre	2.7
Brazil, Cuiaba	2.5
Uganda, Kyadondo County	2.4
Colombia, Manizales	2.4
India, Barshi	2.2
Spain, Cuenca	2.1
Brazil, Sao Paulo	2.0
Thailand, Songkhla	1.9
USA, Puerto Rico	1.9
Chile, Region of Antofagasta	1.8
India, Chennai (Madras)	1.8
Brazil, Fortaleza	1.8

Adapted from Forman et al. [16]

**Table 4:** Comparison of the incidence of previous studies in other states of Brazil and Maranhão

State	Number of cases	Period	Crude incidence rate (100.000 homens/ano)
Maranhão	392	2004-2014	1,18
Bahia [18]	378	1997-2007	0,72
Pará [17]	208	1996-2006	0,46
Pernambuco [6]	88	2007-2012	0,34
Rio de Janeiro [12]	230	2002-2008	0,43

It is believed that incidence of penile cancer is even higher in the state of Maranhão, because a proportion of the affected population seek treatment in the Southeast states, as reported by Favorito et al. [19], where 53.2% of their study participants were from the Northeast, particularly from Maranhão. Maranhão has a geographically isolated capital. Thus, the demand for health services in neighboring states, that are more accessible, is high. Moreover, there are other small care centers for patients with penile cancer in the state of Maranhão; these were not included in our study. Thus, the calculated cumulative incidence in this study is a lower range estimate of the true incidence. Furthermore, the age-standardized estimate of incidence was higher than the crude estimate. This was due to the fact that the population of Maranhão is younger than the standard world population of Segi modified by Doll [16].

A likely explanation for high penile cancer incidence in the state of Maranhão is the high HPV infection rate. An unpublished prospective study (with 57 penile cancer cases from Maranhão) reported an HPV infection rate of over 75% [20]. These data are corroborated by the fact that Maranhão has the highest cervical cancer incidence in Brazil [21], and the pathogenesis of cervical cancer is directly related to HPV infection. Therefore, immunization of male adolescents is pivotal for disrupting the HPV transmission trail. Surprisingly, only three patients were diagnosed with HIV/AIDS (human immunodeficiency virus, acquired immune deficiency syndrome).

**Fig. 3:**
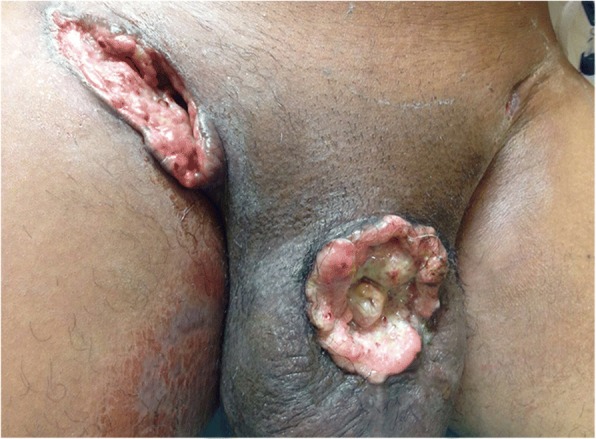
A 42-year-old patient with advanced disease show auto-penectomia and inguinal fistula

The epidemiological and clinical characteristics of the patients in this study were similar to those reported in other studies, particularly those from developing regions whose socioeconomic reality is similar to that of Maranhão [1, 3, 6, 12, 17, 18]. The state of Maranhão has a Human Development Index (HDI) of 0.639, considered the lowest in Brazil. According to IBGE, 71.7% of the families in Maranhão earn less than USD 220.00 a month. In this study, 71% of patients reported being farmers, and 82.1% were from rural areas. When compared with other studies [3, 12], our patients have pretty low smoking rates in the overall population. This would be consistent with the relatively low smoking rates in Maranhão State. Although the average age of 58.5 years is similar to the majority of studies reported in the literature, the Brazil series show a higher frequency of cases in young individuals [18, 19, 22, 23]. This study showed that 19.7% of patients diagnosed with penile cancer were ≤ 40 years of age (Fig. 3). In young people with squamous cell carcinomas, tumor infiltrative growth pattern perineural invasion, and recurrence are more likely [18]. However, the literature and current prognostic indicators do not include the age at diagnosis as an important prognostic marker [8, 18, 24–26].

In terms of the characteristics of the primary tumor, the majority of patients presented with an advanced stage of disease. Approximately 66.4% of tumors were classified as TNM stage T2 or higher. These data are similar to those presented by Couto et al. (63.6%) [6], and slightly higher than those described by Favorito et al. (57.9%) [19]. However, these frequencies are higher than those found in studies conducted in developed countries, such as the United States (45.9 and 50.6%) [26, 27] and Canada (36.4%) [14].

In this case series, penectomy was performed in 93% of patients. Penectomy was partial, total, or involved complete emasculation in 74.7, 17.3, and 1% of cases, respectively. The frequency of penectomy in this series is higher than that reported by Paiva et al. (82%) [18], Koifman et al. (80.9%) [12], Zhu et al. (75.8%) [27], and Chalya et al. (73.9%) [28]. These data demonstrate that patients from Maranhão seek treatment late; hence, they require mutilating treatments. Treatment of penile cancer has a negative impact on welfare in up to 40% of patients and results in psychiatric symptoms in about 50% [29]. The high frequency of more aggressive treatments in this study may contribute to a greater occurrence of psychological and sexual dysfunction.

Among patients who received chemotherapy, 92.3% had advanced, unresectable disease and chemotherapy was palliative. Only two patients received chemotherapy with adjuvant intent and, despite the large proportion of patients with lymph node status N3, only one patient received chemotherapy with neoadjuvant intent. Cisplatin—alone as a radiosensitizing agent or in combination with 5-fluorouracil—and 5-fluorouracil were the most frequently used drugs. The addition of a taxane or ifosfamide was not used as currently recommended [30].

Despite 34.2% of patients having lymph node involvement at the time of undergoing surgical treatment, only 19% of patients treated with radiotherapy were treated with adjuvant intent. The vast majority, 81%, received radiotherapy because of local or regional recurrence and/or ineligibility for surgical rescue. Despite the poor level of evidence for adjuvant radiotherapy, European and American guidelines recommend performing prophylactic radiotherapy for patients at high risk of relapse [31, 32].

The number of patients undergoing lymphadenectomy (41.8%) is much lower than that of patients with tumors classified as ≥ T2 (66.4%) who should, in theory, be submitted to lymph node staging. The lower than expected number of lymphadenectomies is justified by the fact that many patients returned for evaluation only when there was local or regional recurrence.

The presence of recurrences, mainly lymph-node involvement, is the most important prognostic factor for survival in penile cancer [33]. Several possible histological parameters have been associated with recurrences and penile cancer prognosis. These include, histological grade, tumor thickness, vascular embolization, perineural invasion, tumor subtype and, clinical stage [18, 24–26]. In our study, only tumor stage and vascular invasion were significant predictors for disease-free survival. Although, perineural invasion is an important predictor of nodal metastasis [24], our data show no significant increase in risk of disease recurrence.

This study has some limitations, mainly owing to missing data from medical records and the abandonment of treatment by patients. Many patients were rural residents, who had difficulty returning to the capital city to continue treatment. When they again sought medical care, the presence of bulky local and/or regional recurrence—amenable only to palliative treatment—was common. Still, due to the limited patient follow-up, only the deaths of those who died at HCAB were recorded. Finally, the specimens were evaluated by different pathologists, which may be considered as an inherent limitation of multicenter studies.

## Conclusion

With a minimum ASR of 6.1 per 100,000 men and a minimum crude annual incidence rate of 1.18 per 100,000 men, Maranhão has the highest incidence of penile cancer registered in Brazil, and globally, considering data from a single treatment center. The tumors are locally advanced and there is a high frequency among young individuals at the time of diagnosis. Patients generally have low socioeconomic status, making it difficult to complete treatment and attend appropriate follow-up. Therefore, it is necessary to implement measures to enable prevention, early diagnosis, and treatment in order to change this disastrous scenario.

## Abbreviations

ASR: Age-standardized incidence rate
HCAB: Cancer Hospital Aldenora Bello
HDI: Human Development Index
HPV: Human papillomavirus
IARC: International Agency for Research on Cancer
IBGE: Brazilian Institute of Geography and Statistics
